# Persistent Resistant Hypertension Has Worse Renal Outcomes in Chronic Kidney Disease than that Resolved in Two Years: Results from the KNOW-CKD Study

**DOI:** 10.3390/jcm10173998

**Published:** 2021-09-03

**Authors:** Su-Hyun Song, Young-Jin Kim, Hong-Sang Choi, Chang-Seong Kim, Eun-Hui Bae, Curie Ahn, Kook-Hwan Oh, Sue-Kyung Park, Kyu-Beck Lee, Suah Sung, Seung-Hyeok Han, Seong-Kwon Ma, Soo-Wan Kim

**Affiliations:** 1 Department of Internal Medicine, Chonnam National University Medical School, Gwangju 61469, Korea; sudang_@naver.com (S.-H.S.); vfrider@hanmail.net (Y.-J.K.); hongsang38@hanmail.net (H.-S.C.); laminion@hanmail.net (C.-S.K.); baedak76@gmail.com (E.-H.B.); 2 Chonnam National Universitiy Hospital, Gwangju 61469, Korea; 3 Department of Internal Medicine, Seoul National University Hospital, Seoul 03080, Korea; curie@snu.ac.kr (C.A.); ohchris@hanmail.net (K.-H.O.); 4 Department of Preventive Medicine, Seoul National University College of Medicine, Seoul 03080, Korea; suepark@snu.ac.kr; 5 Department of Internal Medicine, Kangbuk Samsung Hospital, Seoul 03181, Korea; kyubeck.lee@samsung.com; 6 Department of Internal Medicine, Nowon Eulji Medical Center, Eulji University, Seoul 01830, Korea; soi@eulji.ac.kr; 7 Department of Internal Medicine, College of medicine, Institute of Kidney Disease Research, Yonsei University, Seoul 03722, Korea; hansh@yuhs.ac

**Keywords:** apparent treatment-resistant hypertension, chronic kidney disease, renal insufficiency, prognosis, end stage renal disease

## Abstract

Apparent treatment-resistant hypertension (ATRH) is closely related to chronic kidney disease (CKD); however, the long-term outcomes and the effects of improvement in ATRH in patients with CKD are not well understood. We evaluated the relationship between the persistence of ATRH and the progression of CKD. This cohort study enrolled 1921 patients with CKD. ATRH was defined as blood pressure above 140/90 mmHg and intake of three different types of antihypertensive agents, including diuretics, or intake of four or more different types of antihypertensive agents, regardless of blood pressure. We defined ATRH subgroups according to the ATRH status at the index year and two years later. The prevalence of ATRH at baseline was 14.0%. The presence of ATRH at both time points was an independent risk factor for end-point renal outcome (HR, 1.41; 95% CI, 1.04–1.92; *p* = 0.027). On the other hand, the presence of ATRH at any one of the time points was not statistically significant. In conclusion, persistent ATRH is more important for the prognosis of renal disease than the initial ATRH status. Continuous follow-up and appropriate treatment are important to improve the renal outcomes.

## 1. Introduction

Hypertension (HTN) is a major risk factor for cardiovascular disease, cerebrovascular accidents, and chronic kidney disease (CKD) [1,2]. It is important to achieve normal blood pressure (BP) through lifestyle modification and pharmacologic treatments. However, many individuals with HTN fail to achieve normal BP despite antihypertensive treatments; this condition is called apparent treatment-resistant hypertension (ATRH). The American Heart Association defines ATRH as BP that is uncontrolled despite the intake of three antihypertensive agents of different classes, including diuretics, or the need for four or more antihypertensive agents to achieve BP control [3,4,5]. A series of large population-based studies and electronic medical record-based studies reported the prevalence of ATRH in the general population to be 13.1–16.3% and 17.5–41.7% in individuals with CKD [6,7,8,9]. In South Korea, the prevalence of ATRH in the general population is 7.9–13.7% [10,11,12].

ATRH is often refractory to treatment and can remain unresolved for many years [13]. The prognosis of ATRH has been actively studied. Recent evidence suggests an association between ATRH and a higher risk of adverse renal outcomes [14,15,16]. However, the long-term outcomes of ATRH in patients with CKD are not well understood. Moreover, no studies have been conducted on the effects of improvement or persistence in ATRH on CKD. Therefore, this study aimed to determine the relationship between renal outcomes and persistence of ATRH in patients with CKD.

## 2. Materials and Methods

### 2.1. Study Participants

The KoreaN cohort study for Outcome in patients with CKD (KNOW-CKD) is a prospective, multicenter, observational cohort study to clarify the natural course, complication profile, and risk factors for the progression of renal disease and complications in patients with CKD. The detailed design and methods of the KNOW-CKD have been published previously [17]. The study was approved by the ethical committee of Seoul National University Hospital. All procedures performed in studies involving human participants were in accordance with the ethical standards of the institutional and/or the national research committee at which the studies were conducted (Seoul National University Hospital: 1104-089-359, Seoul National University Bundang Hospital: B-1106/129-008, Yonsei University Severance Hospital: 4-2011-0163, Kangbuk Samsung Medical Center: 2011-01-076, Seoul St. Mary’s Hospital: KC11OIMI0441, Gil Hospital: GIRBA2553, Eulji General Hospital: 201105-01, Chonnam National University Hospital: CNUH-2011-092, and Pusan Paik Hospital: 11-091) and with the 1964 Helsinki declaration and its later amendments or comparable ethical standards.

**Figure 1:**
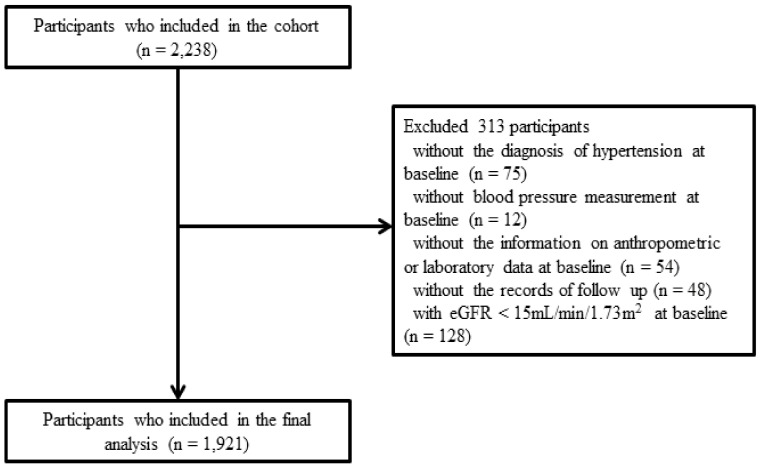
Flow diagram of the study population. Abbreviations: eGFR, estimated glomerular filtration rate.

The KNOW-CKD enrolled 2238 patients with CKD between 2011 and 2016 from nine major university-affiliated tertiary care hospitals throughout South Korea. The participants were followed up until 2020. This study had an ethnically homogenous population group consisting of Koreans aged 20 to 75 years with CKD stages 1 to 5 who were not on dialysis and had not undergone kidney transplantation. A total of 75 participants who did not have information of hypertension were excluded. A further 114 participants were excluded because of inadequate information about their BP or anthropometric or laboratory data at baseline, or lack of follow-up records. A total of 128 participants with an estimated glomerular filtration rate (eGFR) less than 15 mL/min/1.73 m^2^ were also excluded because they were CKD stage 5 [18]. Finally, 1921 participants were included in this study (Figure 1).

### 2.2. Data Collection

The baseline socio-demographic data, including smoking history, current medication, comorbidities, and anthropometric measurements (height, weight, waist and hip circumference, and BP), were collected. Blood samples were obtained after more than 8 h of fasting. A random mid-stream urine sample was collected to measure the urine creatinine, albumin, and protein levels. Serum and random urine creatinine levels were measured using isotope dilution mass spectrometry. Participants visited the clinic 6 months and 12 months after the first visit and annually thereafter. The eGFR was calculated using the Chronic Kidney Disease Epidemiology Collaboration equation [19].

### 2.3. Definition of ATRH and BP Measurement

ATRH was defined as follows: (1) systolic BP ≥ 140 mmHg or diastolic BP ≥ 90 mmHg and intake of three types of antihypertensive agents, including diuretics, or (2) intake of four or more antihypertensive agents, regardless of BP. BP was measured at the nephrology center of each participating hospital at every visit and at the cardiology center at the initial visit and the fourth-year visit. BP was checked using an electronic sphygmomanometer after 5 min of resting in the seated position. The classes of antihypertensive agents were defined according to the JNC8 guidelines [2]. Information about the antihypertensive medications was collected at each visit. Combination pills were considered separately and counted as two or more classes

### 2.4. Study End Point and Definition of the ATRH Subgroups

The primary end point of this study was the progression of CKD, which was defined as a composite of ≥50% reduction in the eGFR from baseline, initiation of long-term hemodialysis or peritoneal dialysis, or kidney transplantation.

Patients were divided into subgroups according to ATRH status at the index year and after two years. Patients with ATRH in the index year but without ATRH in the next two years were assigned to the PRE group. Patients without ATRH in the index year but with ATRH in the next two years were assigned to the POST group. Patients with ATRH both in the index year and after two years were assigned to the BOTH group.

### 2.5. Statistical Methods

The baseline demographic and clinical characteristics were analyzed using the chi-square test or Fisher’s exact test for categorical variables and the Kruskal–Wallis test or one-way analysis of variance for continuous variables. Baseline characteristics are described as numbers and percentages or means with standard deviations. Logistic regression models were used to evaluate the risk factors associated with ATRH. Unadjusted odds ratios (ORs) between ATRH and the predictive factors, including all baseline characteristics, were calculated, and the variables with statistical significance were selected for multivariate logistic regression. Renal outcomes in patients with ATRH were analyzed using the Kaplan–Meier method and Cox proportional hazards model. The results are presented as hazard ratios (HRs) and 95% confidence intervals (CIs). We performed subgroup analysis by sex, age, body mass index (BMI), presence of diabetes mellitus (DM), primary renal outcome, and eGFR using the Cox regression model. These statistical analyses were conducted using the SPSS (version 25.0, SPSS, Inc., Chicago, IL, USA) and R (Version 3.5.2, R Foundation for Statistical Computing, Vienna, Austria) software. All *p*-values were two-sided, and *p* < 0.05 was defined as statistically significant.

## 3. Results

### 3.1. Baseline Characteristics of Study Participants

The baseline characteristics of the population are presented in Table 1. The prevalence of ATRH at baseline was 14.0% (*n* = 268). A higher prevalence of ATRH was observed in men. Additionally, participants with ATRH were older; more likely to be obese; more likely to be past smokers; had lower eGFR, hemoglobin, and high-density lipoprotein levels; and a higher BUN and random urine protein creatinine ratio (PCR). Participants with ATRH were more likely to have a medical history of coronary artery disease, DM, and diabetic kidney disease. Renin-angiotensin system blockers were the most common choice of antihypertensive agents, followed by calcium channel blockers, beta-blockers, diuretics, alpha-blockers, nitrates, and minoxidil. We also present the baseline characteristics of the ATRH subgroups in Supplementary Table S1.

### 3.2. Risk Factors Associated with ATRH

The risk factors associated with ATRH at baseline are shown in Table 2. In the adjusted analysis, male sex (OR, 1.70; 95% CI, 1.24 to 2.32; *p* = 0.001), lower eGFR (OR, 0.98; 95% CI, 0.98 to 0.99; *p* < 0.001), higher BMI (OR, 1.16; 95% CI, 1.11 to 1.20; *p* < 0.001), DM (OR, 1.82; 95% CI, 1.33 to 2.49; *p* < 0.001), higher uric acid OR, 1.15; 95% CI, 1.06 to 1.24; *p* < 0.001), and higher random urine PCR (OR, 1.11; 95% CI, 1.05 to 1.17; *p* < 0.001) were independent risk factors for ATRH.

### 3.3. Association between ATRH Subgroups and Renal Outcome

As shown in Table 3, patients were divided into subgroups according to ATRH status at the index year and after two years. Data of HTN at the index year and after two years were available for 1522 patients. The PRE group comprised 75 patients, the POST group comprised 79 patients, and the BOTH group comprised 105 patients. The control group, who had never been diagnosed with ATRH, was 1263. A total of 467 (31.3%) patients reached the end-point renal outcome.

Adjusted Cox proportional hazard models were used to determine whether ATRH subgroups were independent risk factors for end-point renal outcome (Table 3). In the fully adjusted model, the BOTH group was an independent risk factor for end-point renal outcome (HR, 1.41; 95% CI, 1.04 to 1.92; *p* = 0.027). The PRE group (HR, 1.04; 95% CI, 0.72 to 1.52; *p* = 0.831) and POST group (HR, 1.23; 95% CI, 0.86 to 1.75; *p* = 0.254) were not statistically significant.

**Figure 2:**
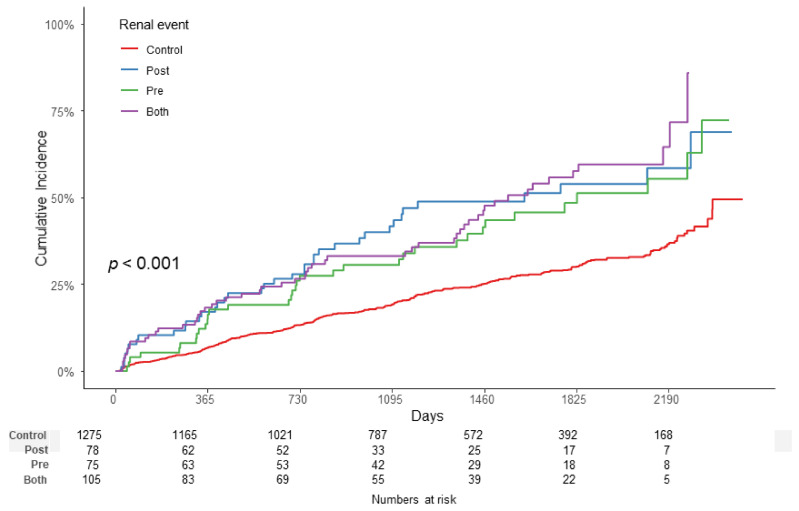
Kaplan–Meier survival curve with log-rank test between the ATRH subgroups. End-point renal outcome occurred more frequently in the ATRH group than in the control group, and among them, the most frequent occurrences were in the BOTH group than in the PRE group and POST group (*p* < 0.001). Abbreviations: ATRH, apparent treatment-resistant hypertension.

Kaplan–Meier survival curves showed statistically significant differences in the incidence of the end-point renal outcome between the ATRH subgroups (*p* < 0.001) (Figure 2). End-point renal outcome occurred more frequently in the ATRH group than in the control group, and among them, the most frequent occurrences were in the BOTH group than in the PRE group and POST group.

### 3.4. Subgroup Analysis

**Figure 3:**
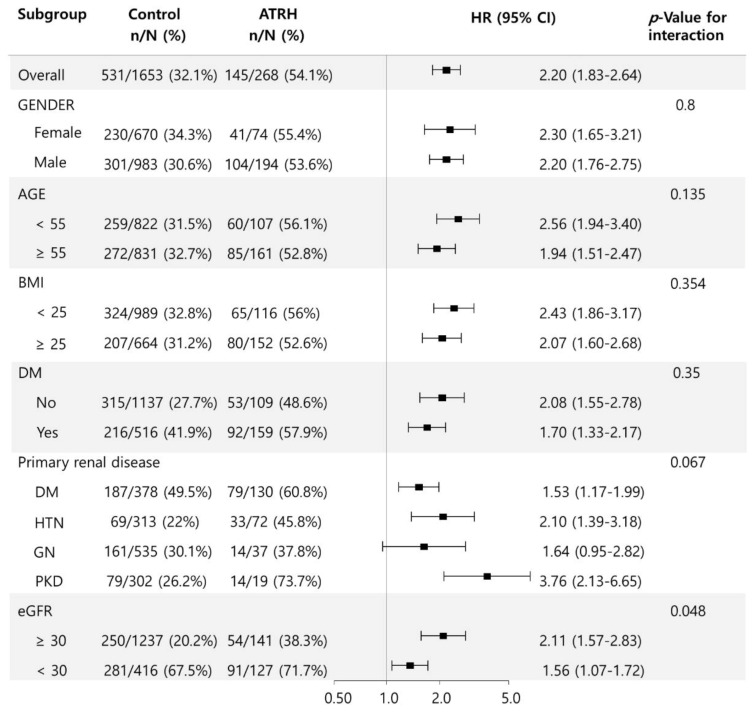
Subgroup analysis for the association between ATRH and end-point renal outcome according to sex, age, BMI, presence of DM, primary renal disease, and eGFR. Abbreviations: ATRH, apparent treatment-resistant hypertension; BMI, body mass index; DM, diabetes mellitus; eGFR, estimated glomerular filtration rate.

We further evaluated the association between ATRH and the end-point renal outcome in several subgroups (Figure 3). There were no significant differences in the outcome according to sex, age, BMI, presence of DM, and the existence of primary renal disease. However, there was significant heterogeneity in the HR for the end-point renal outcome associated with ATRH in the subgroups with eGFR ≥ 30 mL/min/1.73 m^2^ and eGFR < 30 mL/min/1.73 m^2^. eGFR ≥ 30 mL/min/1.73 m^2^ was associated with a significantly higher OR (HR, 2.11; 95% CI, 1.57 to 2.83) than eGFR < 30 mL/min/1.73 m^2^ (HR, 1.56; 95% CI, 1.07 to 1.72) (*p* = 0.048).

## 4. Discussion

The present study showed that unlike the PRE and POST groups, the presence of ATRH at both time points, that is, the BOTH group was an independent risk factor for the end-point renal outcome. This indicates that the persistence of ATRH is more associated with the progression of renal disease than the initial ATRH status. Patients with CKD in whom ATRH resolved within 2 years showed better outcomes than those in whom ATRH persisted. Our study reinforces the importance of continuous patient follow-up. In addition, appropriate treatment can improve renal outcomes. The patients’ BP status, drug compliance, and lifestyle factors should be monitored and discussed with the patient regularly.

The KNOW-CKD is the largest cohort study to investigate the clinical course and complications in patients with CKD in South Korea. In this study, the prevalence of ATRH was relatively lower than that in cohorts of CKD patients from other countries; it was more similar to that in the general hypertensive population. In a comparative study, the prevalence of ATRH was higher in the European population and lower in cohorts from North America, high-income Asian countries, and Australia [20]. Moreover, other studies evaluated the prevalence of ATRH in patients with stage 3–5 CKD [7,21]. However, this study included a relatively high proportion of participants with good baseline kidney function. A total of 657 (34.2%) of the 1921 participants had a baseline eGFR > 60 mL/min/1.73 m^2^, and 303 (15.8%) had a baseline eGFR > 90 mL/min/1.73 m^2^. These patients were enrolled because of proteinuria or structural abnormalities of the kidney, such as autosomal dominant polycystic kidney disease or glomerulonephropathy. Furthermore, the homogenous ethnic composition of the study population might also be the cause for the difference in prevalence.

Previous studies have suggested that advanced age, male sex, lower baseline renal function, a higher degree of proteinuria/albuminuria, DM, and a higher BMI are independently associated with the prevalence of ATRH [8,22,23]. Our study showed similar findings; male sex, lower baseline renal function, a higher degree of proteinuria, presence of DM, and a higher BMI were independent risk factors for ATRH. These results emphasize that in males who have DM, proteinuria, or are obese, constant management is necessary to prevent persistent ATRH.

Several large studies have shown an association between the severity of HTN and progression of CKD. In a cohort of more than 470,000 patients with HTN followed up over 5 years, ATRH was an independent risk factor for end-stage renal disease (ESRD) [24]. There are some potential mechanisms by which ATRH could be a major factor in the progression of CKD. Normally, the glomerular capillary loops are protected from elevated systemic arterial pressures by an autoregulatory mechanism. However, in hypertensive patients, chronically elevated systemic arterial pressure causes remodeling of the afferent arterioles and reduces their ability. Finally, this leads to glomerular hypertension, sclerosis, and progressive loss of kidney function [25,26]. In our study, the persistence of ATRH showed CKD progression compared to ATRH that resolved. In addition, our subgroup analysis showed that patients with early CKD had a higher effect of ATRH on adverse renal outcomes than those with advanced CKD. This result reinforces that management of ATRH should be initiated from the early CKD stage.

Our study had some limitations. First, the study population was enrolled only from tertiary care hospitals, and we could not enroll patients with hypertension from primary clinics. The prevalence of ATRH might be underestimated in tertiary care hospitals. Second, drug compliance was not measured accurately. Poor compliance to medications is a major cause of failure to achieve normal BP [27]. However, our study also had several strengths. First, to our knowledge, this is the first study that showed the association between the persistence of ATRH and renal adverse outcomes. Second, this study had a large sample size, comprehensive CKD diseases, and long-term follow-up of disease outcomes.

## 5. Conclusions

In conclusion, the persistence of ATRH is associated with adverse renal outcomes. This finding suggests that continuous follow-up and appropriate treatment are important to prevent the progression of renal disease.

## Figures and Tables

**Table 1:** Clinical characteristics of the study population.

Variable	Total Population (*n* = 1921)		*p*-Value
	No ATRH (*n* = 1653)	ATRH † (*n* = 268)	
Age, year (average ± SD)	53.4 ± 12.3	56.3 ± 12.4	<0.001
Male gender, *n* (%)	983 (59.5%)	194 (72.4%)	<0.001
Education:			0.791
No education or illiteracy	25 (1.5%)	5 (1.9%)	
Elementary school graduate	173 (10.5%)	32 (11.9%)	
Middle school graduate	206 (12.5%)	28 (10.4%)	
High school graduate	565 (34.2%)	89 (33.2%)	
More than college graduate	684 (41.4%)	114 (42.5%)	
Employment status, *n* (%)	1223 (74.0%)	190 (70.9%)	0.322
Smoking status:			<0.001
Never	909 (55.5%)	112 (41.8%)	
Ex-smoker	481 (29.1%)	117 (43.7%)	
Current smoker	263 (15.9%)	39 (14.6%)	
Primary renal disease:			<0.001
Diabetic kidney disease	378 (22.9%)	130 (48.5%)	
Hypertensive renal disease	313 (18.9%)	72 (26.9%)	
Glomerulonephropathy	535 (32.4%)	37 (13.8%)	
Tubulointerstitial nephropathy	12 (0.7%)	0 (0.0%)	
Polycystic kidney disease	302 (18.3%)	19 (7.1%)	
Other kidney disease	113 (6.8%)	10 (3.7%)	
Coronary artery disease, *n* (%)	97 (5.9%)	32 (11.9%)	<0.001
DM, *n* (%)	516 (31.2%)	159 (59.3%)	<0.001
BMI, Kg/m^2^ (average ± SD)	24.4 ± 3.3	26.2 ± 3.7	<0.001
Serum creatinine, mg/dL (average ± SD)	1.7 ± 1.1	2.5 ± 1.4	<0.001
eGFR, mL/min/1.73 m^2^ (average ± SD)	54.9 ± 30.8	37.0 ± 24.0	<0.001
BUN, mg/dL (average ± SD)	27.0 ± 15.0	38.2 ± 18.8	<0.001
Random urine ACR, mg/gCr (average ± SD)	781.3 ± 1276.0	1621.5 ± 2008.3	<0.001
Random urine PCR, g/gCr	1.1 ±1.9	2.4 ± 3.1	<0.001
Serum hemoglobin, g/dL (average ± SD)	12.9 ± 2.0	12.3 ± 2.1	<0.001
Serum albumin, g/dL (average ± SD)	4.2 ± 0.4	4.1 ± 0.5	<0.001
Serum uric acid, mg/dL (average ± SD)	6.9 ± 1.9	7.9 ± 2.0	<0.001
Na, mmol/L (average ± SD)	140.8 ± 2.4	140.8 ± 2.8	0.702
K, mmol/L (average ± SD)	4.6 ± 0.6	4.8 ± 0.6	<0.001
Total cholesterol, mg/dL (average ± SD)	173.6 ± 38.4	176.3 ± 42.7	0.330
LDL, mg/dL (average ± SD)	96.8 ± 31.2	96.0 ± 31.5	0.706
HDL, mg/dL (average ± SD)	49.9 ± 15.4	44.4 ± 14.2	<0.001
Triglyceride, mg/dL (average ± SD)	151.4 ± 92.1	189.5 ± 119.8	<0.001
Systolic blood pressure, mmHg (average ± SD)	126.5 ± 15.2	136.7 ± 18.6	<0.001
Diastolic blood pressure, mmHg (average ± SD)	76.4 ± 10.6	80.0 ± 13.4	<0.001
Taking aspirin, *n* (%)	418 (25.3%)	117 (43.7%)	<0.001
Number of anti-hypertensive agents	1.7 ± 0.9	4.1 ± 0.7	<0.001
ACE inhibitor	160 (9.7%)	53 (19.8%)	<0.001
Angiotensin receptor blocker	1295 (78.3%)	251 (93.7%)	<0.001
Diuretics	392 (23.7%)	216 (80.6%)	<0.001
Thiazide	163 (9.9%)	66 (24.6%)	<0.001
Loop diuretics	207 (12.5%)	144 (53.7%)	
Potassium sparing diuretics	22 (1.3%)	6 (2.2%)	
Beta blocker	277 (16.8%)	225 (84.0%)	<0.001
Nondihydropyridine CCB	28 (1.7%)	17 (6.3%)	<0.001
Dihydropyridine CCB	558 (33.8%)	244(91.0%)	<0.001
Nitrate	25 (1.5%)	27 (10.1%)	<0.001
Alpha blocker	44 (2.7%)	57 (21.3%)	<0.001
Minoxidil	2 (0.1%)	11 (4.1%)	<0.001

Abbreviations: ATRH, apparent treatment-resistant hypertension; SD, standard deviation; DM, diabetes mellitus; BMI, body mass index; eGFR, estimated glomerular filtration rate; ACR, albumin creatinine ratio; PCR, protein creatinine ratio; Na, sodium; K, potassium; LDL, low-density lipoprotein; HDL, high-density lipoprotein; ACE, angiotensin converting enzyme; CCB, calcium channel blocker; † ATRH is defined as systolic blood pressure ≥ 140 mmHg or diastolic blood pressure ≥ 90 mmHg with intake of three anti-hypertensive agents, including diuretics, or more than four anti-hypertensive agents.

**Table 2:** Risk factors associated with ATRH.

Variable	Unadjusted Odds Ratio (95% CI)	*p*-Value	Adjusted Odds Ratio (95% CI)	*p*-Value
Age (per 1 year change)	1.02 (1.01–1.03)	<0.001	1.00 (0.99, 1.01)	0.838
Male sex (reference: female)	1.79 (1.34–2.38)	<0.001	1.70 (1.24–2.32)	0.001
eGFR (per 1 mL/min/1.73 m^2^ change)	0.98 (0.97–0.98)	<0.001	0.98 (0.98–0.99)	<0.001
BMI (per 1 kg/m^2^ change)	1.15 (1.11–1.20)	<0.001	1.16 (1.11–1.20)	<0.001
Diabetes	3.21 (2.47–4.19)	<0.001	1.82 (1.33–2.49)	<0.001
Coronary artery disease	2.18 (1.43–3.32)	<0.001	1.24 (0.78–1.97)	0.764
Uric acid (per 1 mg/dL change)	1.28 (1.20–1.37)	<0.001	1.15 (1.06–1.24)	<0.001
LDL (per 1 mg/dL change)	1 (1.00–1.00)	0.703	1 (1.00–1.01)	0.485
Na (per 1 mmol/L change)	0.99 (0.94–1.04)	0.665	1.01 (0.96–1.07)	0.635
Random urine PCR (per 1 g/gCr)	1.21 (1.16–1.27)	<0.001	1.11 (1.05–1.17)	<0.001

Abbreviations: ATRH, apparent treatment-resistant hypertension; CI, confidence interval; eGFR, estimated glomerular filtration rate; BMI, body mass index; LDL, low density lipoprotein; Na, sodium; PCR, protein creatinine ratio.

**Table 3:** Hazard ratios for renal outcome according to the ATRH subgroups.

		PRE Group	POST Group	BOTH Group
**Unadjusted**	**HR (95% CI)**	1.85 (1.31–2.62)	2.11 (1.50–2.98)	2.30 (1.72–3.08)
	***p*** **-Value**	< 0.001	< 0.001	< 0.001
**Model 1**	**HR (95% CI)**	1.87 (1.32–2.65)	2.11 (1.49–2.98)	2.32 (1.73–3.12)
	***p*** **-Value**	< 0.001	< 0.001	< 0.001
**Model 2**	**HR (95% CI)**	1.44 (1.01–2.06)	1.70 (1.19–2.42)	2.08 (1.53–2.83)
	***p*** **-Value**	0.043	0.003	<0.001
**Model 3**	**HR (95% CI)**	1.04 (0.72–1.52)	1.23 (0.86–1.75)	1.41 (1.04–1.92)
	***p*** **-Value**	0.831	0.254	0.027

Abbreviation: ATRH, apparent treatment-resistant hypertension; HR, hazard ratio; CI, confidence interval. **Model 1**: Adjusted for age and gender. **Model 2**: Adjusted for Model 1 and additionally for diabetes, body mass index, uric acid, low-density lipoprotein, coronary artery disease, and smoking status. **Model 3**: Adjusted for Model 2 and additionally for estimated glomerular filtration rate and random urine protein creatinine ratio.

## Supplementary Materials

The following are available online at https://www.mdpi.com/article/10.3390/jcm10173998/s1, Supplementary Table S1. Clinical characteristics of ATRH subgroups.
