# Supplementary material for: Persistent Resistant Hypertension Has Worse Renal Outcomes in Chronic Kidney Disease than that Resolved in Two Years: Results from the KNOW-CKD Study
Source: J Clin Med. 2021 Sep 3;10(17):3998. doi: 10.3390/jcm10173998 (PMC8432533; doi:10.3390/jcm10173998)
Supplement: Supplementary file 1 [file jcm-10-03998-s001.zip › jcm-1351486-supplementary.pdf]

Supplementary Table S1. Clinical characteristics of ATRH subgroups.

Variables	Control (n = 1263)	PRE group (n = 75)	POST group (n = 79)	BOTH group (n = 105)	*p*-value
Age, year (average ± SD)	53.1 ± 12.0	57.2 ± 13.0	56.3 ± 11.3	57.1 ± 12.1	<0.001
Male gender, n (%)	745 (59.0%)	52 (69.3%)	48 (60.8%)	79 (75.2%)	0.004
Education					0.290
No education or illiteracy	19 (1.5%)	2 (2.7%)	1 (1.3%)	3 (2.9%)	
Elementary school graduate	129 (10.2%)	8 (10.7%)	11 (13.9%)	14 (13.3%)	
Middle school graduate	145 (11.5%)	7 (9.3%)	16 (20.3%)	9 (8.6%)	
High school graduate	434 (34.4%)	28 (37.3%)	28 (35.4%)	35 (33.3%)	
More than college graduate	536 (42.4%)	30 (40%)	23 (29.1%)	44 (41.9%)	
Employment status, n (%)	963 (76.2%)	58 (77.3%)	60 (75.9%)	77 (73.3%)	0.914
Smoking status					0.001
Never	707 (56.0%)	32 (42.7%)	38 (48.1%)	41 (39%)	
Ex-smoker	356 (28.2%)	31 (41.3%)	29 (36.7%)	49 (46.7%)	
Current smoker	200 (15.8%)	12 (16.0%)	12 (15.2%)	15 (14.3%)	
Primary renal disease					<0.001
Diabetic kidney disease	234 (18.5%)	30 (40.0%)	34 (43.0%)	44 (41.9%)	
Hypertensive renal disease	246 (19.5%)	16 (21.3%)	20 (25.3%)	41 (39.0%)	
Glomerulonephropathy	439 (34.8%)	14 (18.7%)	16 (20.3%)	11 (10.5%)	
Tubulointerstitial nephropathy	11 (0.9%)	0 (0.0%)	0 (0.0%)	0 (0.0%)	
Polycystic kidney disease	244 (19.3%)	11 (14.7%)	7 (8.9%)	4 (3.8%)	
Other kidney disease	89 (7.0%)	4 (5.3%)	2 (2.5%)	5 (4.8%)	
Coronary artery disease, n (%)	59 (4.7%)	6 (8.0%)	9 (11.4%)	17 (16.2%)	<0.001
DM, n (%)	348 (27.6%)	38 (50.7%)	43 (54.4%)	58 (55.2%)	<0.001
BMI, Kg/m^2 (average ± SD)	24.4 ± 3.3	25.8 ± 3.5	25.6 ± 3.3	26.4 ± 3.9	<0.001
Serum creatinine, mg/dL (average ± SD)	1.5 ± 0.8	1.9 ± 0.7	1.8 ± 0.8	2.0 ± 0.8	<0.001
eGFR, mL/min/1.73m^2 (average ± SD)	58.4 ± 29.6	43.8 ± 22.2	46.6 ± 23.9	41.4 ± 21.7	<0.001
BUN, mg/dL (average ± SD)	24.8 ± 12.7	30.0 ± 11.5	28.9 ± 12.2	34.3 ± 16.3	<0.001
Random urine ACR, mg/gCr (average ± SD)	577.2 ± 908.7	1114.7 ± 1590.0	1072.9 ± 1288.6	984.5 ± 1200.1	<0.001
Random urine PCR, g/gCr	0.8 ± 1.3	1.6 ± 2.3	1.5 ± 1.8	1.4 ± 1.7	<0.001
Serum hemoglobin, g/dL (average ± SD)	13.2 ± 1.9	12.9 ± 2.0	12.4 ± 1.9	12.8 ± 1.9	0.003
Serum albumin, g/dL (average ± SD)	4.3 ± 0.3	4.2 ± 0.5	4.1 ± 0.4	4.2 ± 0.4	0.002
Serum uric acid, mg/dL (average ± SD)	6.9 ± 1.9	7.7 ± 2.0	7.2 ± 1.7	7.5 ± 1.9	<0.001
Na, mmol/L (average ± SD)	140.9 ± 2.3	141.4 ± 2.2	141.4 ± 2.5	140.5 ± 3.0	0.022
K, mmol/L (average ± SD)	4.6 ± 0.5	4.6 ± 0.6	4.7 ± 0.6	4.7 ± 0.6	0.024
Total cholesterol, mg/dL (average ± SD)	151.0 ± 91.5	176.5 ± 42.4	164.2 ± 34.6	174.9 ± 37.2	0.089
LDL, mg/dL (average ± SD)	97.0 ± 30.1	96.4 ± 33.8	87.5 ± 26.5	95.7 ± 29.1	0.026
HDL, mg/dL (average ± SD)	50.4 ± 15.2	48.1 ±14.3	47.3 ± 16.2	43.5 ± 11.7	<0.001
Triglyceride, mg/dL (average ± SD)	151.0 ± 91.5	180.8 ± 101.8	158.6 ± 116.2	202.4 ± 135.0	<0.001
Systolic blood pressure, mmHg (average ± SD)	125.0 ± 14.3	135.4 ± 19.2	133.8 ± 12.8	133.2 ± 15.9	<0.001
Diastolic blood pressure, mmHg (average ± SD)	76.0 ± 10.2	80.5 ± 12.8	78.8 ± 9.2	78.3 ± 13.3	<0.001
Taking aspirin, n (%)	313 (24.8%)	34 (45.3 %)	31 (39.2%)	47 (44.8%)	<0.001
Number of anti-hypertensive agents	1.6 ± 0.9	3.9 ± 0.6	2.5 ± 0.8	4.2 ± 0.7	<0.001
ACE inhibitor	124 (9.8%)	14 (18.7%)	6 (7.6%)	22 (21.0%)	<0.001
Angiotensin receptor blocker	1002 (79.3%)	70 (93.3%)	63 (79.7%)	98 (93.3%)	<0.001
Diuretics					<0.001
Thiazide	117 (9.3%)	23 (30.7%)	17 (21.5%)	29 (27.6%)	<0.001

Variables	Control (n = 1263)	PRE group (n = 75)	POST group (n = 79)	BOTH group (n = 105)	*p*-value
Loop diuretics	125 (9.9%)	37 (49.3%)	15 (19.0%)	51 (48.6%)	
Potassium sparing diuretics	18 (1.4%)	0 (0.0%)	1 (1.3%)	4 (3.8%)	
Beta blocker	183 (14.5%)	58 (77.3%)	36 (45.6%)	93 (88.6%)	<0.001
Nondihydropyridine CCB	21 (1.7%)	6 (8.0%)	0 (0.0%)	6 (5.7%)	0.002
Dihydropyridine CCB	389 (30.8%)	64 (85.3%)	52 (65.8%)	98 (93.3%)	<0.001
Nitrate	16 (1.3%)	9 (12.0%)	2 (2.5%)	11 (10.5%)	<0.001
Alpha blocker	31 (2.5%)	12 (16.0%)	4 (5.1%)	27 (25.7%)	<0.001
Minoxidil	1 (0.1%)	1 (1.3%)	0 (0.0%)	7 (6.7%)	<0.001

Abbreviations: ATRH, apparent treatment resistant hypertension; SD, standard deviation; DM, diabetes mellitus; BMI, body mass index; eGFR, estimated glomerular filtration rate; ACR, albumin creatinine ratio; PCR, protein creatinine ratio; Na, sodium; K, potassium; LDL, low density lipoprotein; HDL, high density lipoprotein; ACE, angiotensin converting enzyme; CCB, calcium channel blocker.

† ATRH subgroups are defined according to the ATRH status at the index year and after two years. Patients with ATRH in the index year but without ATRH in the next two years were assigned to the PRE group. Patients without ATRH in the index year but with ATRH in the next two years were assigned to the POST group. Patients with ATRH both in the index year and after two years were assigned to the BOTH group. Patients without ATRH in both index year and next two years were assigned to the Control group.
